# Biochemical Features of the Cry3A Toxin of *Bacillus thuringiensis* subsp. *tenebrionis* and Its Toxicity to the Red Imported Fire Ant *Solenopsis invicta*

**DOI:** 10.3390/microorganisms13020371

**Published:** 2025-02-08

**Authors:** Lee A. Bulla, Jr.

**Affiliations:** Department of Biological Sciences, The University of Texas at Dallas, Richardson, TX 75080-3021, USA; bulla@utdallas.edu

**Keywords:** *Bacillus thuringiensis* subsp. *tenebrionis*, Cry3A, red imported fire ant

## Abstract

Bioinsecticides based on the bacterium *Bacillus thuringiensis* (Bt) are widely used as safe alternatives to chemical insecticides. The insecticidal activity of Bt is occasioned by a protein toxin contained in parasporal crystals (Cry proteins) that are synthesized and laid down alongside the endospore during sporulation. The specificity of toxin action is associated with the subspecies of Bt and the individual Cry toxins they produce. Although a number of commercial Bt formulations are available to control moths, mosquitoes and beetles, there are none that control the red imported fire ant (RIFA) *Solenopsis invicta*. The present report is the first to describe the insecticidal activity of the Cry3A protein toxin, produced by *Bacillus thuringiensis* subsp. *tenebrionis* (Btt), against the RIFA as well as some of its key biochemical properties. Currently available commercial formulations of Btt are designed to control beetles such as the Colorado potato beetle, not ants. The Cry3A toxin (MW ~66 kDa) is embedded in a larger polypeptide (protoxin, MW ~73 kDa) and is released from the toxin enzymatically. Once activated, it can be administered to the RIFA as a soluble protein that most likely binds to an attendant receptor in the epithelial cells that line the wall of the larval ventriculus, killing the insect. Properly customized, the Cry3A toxin is a potential candidate for fire ant control.

## 1. Introduction

*Bacillus thuringiensis* subsp. *tenebrionis* (Btt), a Gram-positive spore-forming bacterium, produces a complex of crystalline protein inclusion bodies (also known as parasporal crystals) that are toxic to larvae of a variety of coleopteran insects (beetles). The bacterium was first isolated from an infected pupa of the yellow mealworm *Tenebrio molitor* (Coleoptera: Tenebrionidae) [1]. Synthesis of the parasporal crystal protein (Cry protein) is uniquely genetically orchestrated with development and production of the endospore. The protein is composed exclusively of a single protoxin polypeptide (MW ~73 kDa) that constitutes 30–40% of the cell dry weight. Enzyme activation of the protoxin generates a single monomeric toxin (MW ~66 kDa). Commercial formulations of Btt containing spores and insecticidal crystal proteins (Cry toxins) are successful in controlling the yellow mealworm, Colorado potato beetle, southern corn rootworm, elm leaf beetle, tuber flea beetle and alfalfa weevil, among others. Transgenic potato plants containing the *cry3a* gene also are extremely effective in protecting the plants from the Colorado potato beetle [2,3]. For the past several decades, my laboratory has been engaged in determining how Bt-produced Cry toxins kill various insects including moths, mosquitoes and beetles, which are economically important to agriculture and public health. Of particular interest is the Cry3A (also known as Cry3Aa) protein toxin (UniProtKB P0A379) produced by Btt. To date, all published studies have concentrated on the effect of the Cry3A toxin on beetles, particularly the Colorado potato beetle. The present report is the first description of the insecticidal activity of Cry3A against the red imported fire ant (RIFA) *Solenopsis invicta* Buren.

The RIFA is an agriculturally and medically important pest harmful to domestic animals, wildlife and humans. The fire ant typically causes painful stings. Its venom contains pyridine, piperidine and piperideine alkaloids [4] that can cause necrosis at the sting site generating significant medical issues such as allergen hypersensitivity [5] and anaphylaxis [6,7]. Besides the impact on human health, deleterious effects on social and mental well-being also have been reported [8,9]. Fire ants threaten obligate ground dwelling species and young animals of all species. Indeed, the RIFA is believed to be responsible for the decline of several native species. High densities of fire ants cause damage to roads, pastures and electrical and mechanical equipment. The RIFA builds mounds in pastures, fields and cultivated crops. In urban areas, it nests in lawns, under edges of sidewalks, foundations, patio slabs and concrete and asphalt driveways. Because of the fire ant’s attraction to electricity, it causes destruction of HVAC units, water well equipment, outdoor lighting, circuit breakers and the like, creating hazardous conditions. Proliferation of the RIFA has gone largely unchecked because of the absence of predators, pathogens and parasites that control its numbers in its native environment. The appearance of multiple queen colonies has made control of fire ant populations increasingly difficult. An effective compound and method of control of fire ant populations that is safe to users and consumers is urgently needed.

Presented here are some of the key biochemical features of the Cry3A toxin and protoxin from which it is derived and the insecticidal activity of both native and recombinant toxins to the RIFA. The Cry3A toxin is embedded in a protoxin which occurs as a single repeating subunit in the parasporal crystals deposited alongside the endospore during sporulation. Following ingestion by *S*. *invicta* fourth-instar larvae, an N-terminal segment of the protoxin is cleaved by proteases in the blind midgut, generating active toxin which is transferred to the ventriculus that is lined with ciliated columnar epithelial cells [10]. The *cry3a* gene encodes the Cry3A toxin, a putative first messenger cell-death signaling protein which most likely binds directly to a bitopic cadherin G protein-coupled receptor (GPCR) localized in the epithelial cells of the ventriculus. In turn, the second messenger cAMP is activated, promoting a cell-death signaling pathway. Bitopic cadherin GPCRs have been described for the Cry1A and Cry4B toxins that kill moths and mosquitoes, respectively [11,12]. A single binding protein (MW ~144 kDa) from *T. molitor* specific for the Cry3A toxin of Btt has been identified and purified [13]. The Cry3A toxin binds the 144 kDa protein (K_d_ = 17.5 nM) and kills *T. molitor* larvae (LC_50_ = 21 µg/cm^2^). Soluble activated Cry3A toxin—both native and recombinant—is highly potent to RIFA larvae maintained under laboratory conditions and native toxin is quite effective against fire ants living in field mounds. Expression vectors like the one described herein can be used to produce novel surrogate hosts which are engineered to express the encoded Cry3A protein either transiently upon induction or constitutively.

Chemical insecticides are prominent in managing and controlling the RIFA and other ant species. Most of the commercial products available are bait formulations that contain various compounds such as fenoxycarb (carbamate ester, a juvenile hormone mimic), S-Methoprene (a juvenile hormone analog and insect growth regulator), pyriproxyfen (pyridine-based juvenile hormone mimic), indoxacarb (an organochlorine), acephate (organic thiophosphate), hydramethylnon (hydrazone, a class of azomethines), spinosad (unspecified molecular formula primarily used to control head lice), abamectin (member of the avermectin family), metaflumizone (a stilbenoid and a semicarbazone) and borax (a mineral also known as sodium borate or borax decahydrate). Other compounds that are marketed for the fire ant include amidinohydrazone (a synthetic guanyl hydrazone derivative otherwise referred to as benzhydrazone), neonicotinoid (imidacloprid, a class of neuroactive insecticides modeled after nicotine); organophospates (a class of organophosphorus compounds), phenylpyrazol (contains a central pyrazole ring with a phenyl group) and pyrethroids (synthetic esters derived from naturally occurring pyrethrins). Although some of these compounds are marketed specifically for the RIFA, most are broad-spectrum insecticides and are potential health and/or environmental hazards [14]. Insect resistance to many of these compounds also is a worldwide problem. Most notably, as the World Health Organization [15] points out, “… chemical pesticides are potentially toxic to other organisms, including humans, and need to be used safely and disposed of properly. They are among the leading causes of death by self-poisoning, and this burden is felt disproportionately in low- and middle-income countries”. Because of the problems associated with heavy use of chemical insecticides, current insect control practices combine chemicals with alternative biocontrol measures involving incorporation of natural enemies such as (i) parasites like the phorid fly; (ii) microsporidial and viral pathogens; and (iii) predators including armadillos, spiders, insects, birds and horned lizards. So far, success with these alternative approaches has been mixed and limited. The disadvantages of using natural enemies are (i) incomplete repression of the target community, (ii) negative impacts on non-target and endangered species and (iii) difficulty in establishing appropriate management strategies. One alternative that has not been considered seriously is the use of a *Bacillus thuringiensis* (Bt)-based insecticide for controlling fire ant populations. Bt-based insecticides are used universally to control a broad range of pest insects. To wit, commercial formulations containing different Cry toxins are marketed exclusively to control moths, beetles and mosquitoes. However, until now, a Cry toxin with specificity for the fire ant has not been realized.

## 2. Materials and Methods

### 2.1. Isolation of and Culture Conditions for Btt

Btt strain UTD-001 (NRRL No. B-30356) was originally isolated from soil samples near red ant fire mounds in a pasture at Cinnabar Ranch, Tioga, TX. A 1-g sample of soil was mixed with 100 mL of Luria Broth (LB) [16] buffered with 0.25 M NaOAc, pH 6.8, and incubated with shaking (200 rpm) overnight at 30 °C. A 1.5 mL aliquot of the sample was removed and heat-shocked at 80 °C with agitation in a 50 mL conical tube for 3 min. Aliquots of 10, 20 and 100 µL then were plated on Luria Agar (LA) plates [16] and incubated overnight at 30 °C. Isolated colonies were selected and cultured in a modified GYS medium containing the following components (per liter): glucose (2.0 g), (NH_4_)_2_SO_4_ (2.0 g), CaCl_2_ (0.08 g), MgSO_4_ (0.20 g), MnSO_4_ (0.05 g), citric acid (1.5 g) and yeast extract (2.0 g). The pH was adjusted to 7.5 with KOH.

### 2.2. Purification of Native Cry3A Toxin

The Btt strain UTD-001 was grown in 1 L of modified GYS medium for 4–6 days until most of the cells were lysed. The medium containing the lysed cells was centrifuged for 20 min at 7500 rpm, the supernatant discarded, and the pellet transferred to a 40 mL centrifuge tube and resuspended in 1M NaCl, centrifuged at 15,000 rpm, and washed 2× with dH_2_O. Parasporal crystals were separated from spores by buoyant density centrifugation and washed in PBS buffer for storage at −20 °C or for immediate use. The crystals were solubilized in 3.3 M NaBr and 50 mM phosphate buffer (pH 7.0) containing 1 mM PMSF. Immobilized papain beads (Thermo Scientific™, Waltham, MA, USA) were activated according to the manufacturer’s instructions and added to the solubilized crystals containing native Cry3A protoxin (MW ~73 kDa), a single repeating subunit, which was cleaved to Cry3A toxin (MW ~66 kDa). Cleavage was arrested by addition of TLCK. The toxin was dialyzed against buffer containing 50 mM Na_2_CO_3_ (pH 10) containing 1 mM EDTA and 5% glycerol and purified by anion exchange and gel filtration chromatography using an ÄKTA Fast Protein Liquid Chromatography system (Cytiva). Fractions containing purified Cry3A were pooled, concentrated by centrifugation, quantified by the Bradford assay [17] and visualized by SDS-PAGE. Purification yield was 35–40%. All procedures were performed under non-reducing conditions. Aliquots of the purified Cry3A toxin were stored at −80 °C.

### 2.3. Cloning and Expression of the cry3a Gene

A 1.8 kb PCR product of total UTD-001 DNA with *Taq*-created A overhangs was excised from a low-melt agarose gel and ligated, using T4 DNA ligase, into linearized pCR2.1 with T overhangs to produce the recombinant pCCRY3 plasmid (Invitrogen™ TA Cloning Kit, Waltham, MA, USA). Following PCR amplification using appropriate primers, the *cry3a* gene was analyzed in a 1% agarose gel and visualized by EtBr staining. The amplicon was excised from the gel and purified. *Escherichia coli* TOPO10 and BL21 (DE3) strains were used for DNA manipulation and *cry3a* gene expression, respectively. The purified *cry3a* gene was cloned in pENTER/D-TOPO and then subcloned in pDEST-17 and transformed into *E. coli* BL21 for expression. The *cry3a* gene was under the control of the T7 promoter. Recombinant protein purification was accomplished by growing the BL21 cells overnight in LA broth containing ampicillin (100 µg/mL). Gene expression was induced by adding IPTG (0.5 nM) and harvesting the cells by centrifugation several hours later. Identification of the recombinant Cry3A toxin was accomplished by Western blot analysis using anti-Cry3A antibody and visualizing with rabbit anti-goat antibody coupled with horseradish peroxidase. Purification yield was 15–20%. The expressed toxin (581 amino acids; MW = 65.792 kDa) is devoid of the first 63 amino acids present in the native protoxin POA379 CR3AA-BACTT described earlier [18], which is cleaved by papain at ^63^KD^64^.

### 2.4. Fire Ant Maintenance and Bioassays

Fragments of fire ant colonies were collected from mounds in the field (Cinnabar Ranch, Tioga, TX, USA) and transferred directly to the laboratory in Fluon^®^-coated 2-gallon plastic buckets (AGC Chemicals, Exton, PA, USA). Maintenance of the ant colonies was performed according to previously published protocols [19], with modification. The colonies were kept in the laboratory in screened plastic Petri dishes (25 × 150 mm) lined with Whatman 1005-150 filter circles and fed crickets (Armstrong’s Cricket Farm, West Monroe, LA, USA) and 10% sucrose applied to small cotton strips and balls placed in the dishes. The dishes were stationed in Nalgene^®^ lab trays and held in a climate-controlled incubator (27 °C; 50% RH). Separate colonies of worker ants were examined for the effect of the toxin (200 µg/mL) which was mixed in a 10% solution of sucrose in dH_2_O. The bioassays were based on a protocol established for the tobacco hornworm [20]. Prior to the tests, the worker ant colonies were held without food for 5 days after which 20 workers were placed in 300 mL cups whose inner surface at the top was treated with Fluon^®^ to prevent ants from escaping. Toxin preparations were placed in the cups for only the first 24 h and then removed and replaced with 10% sucrose in dH_2_O without toxin. Percent mortality was recorded on days 1, 2, 3, 6, 8, 10 and 14 by recording the number of viable and dead ants. Mortality (%) was calculated by the ratio of (dead ants/total ants) × 100. The lethal concentration required to kill 50% (LC_50_) of the ants was determined by the percentage of surviving ants exposed to various concentrations (0–100 ng/mL) of soluble Cry3A toxin (viable ants/total ants × 100). Bioassay results were analyzed using GraphPad 10.4.1 (627) and represent the mean ± SD of six independent experiments replicated five times.

## 3. Results

### 3.1. Domain Structure of the Cry3A Toxin

**Figure 1:**
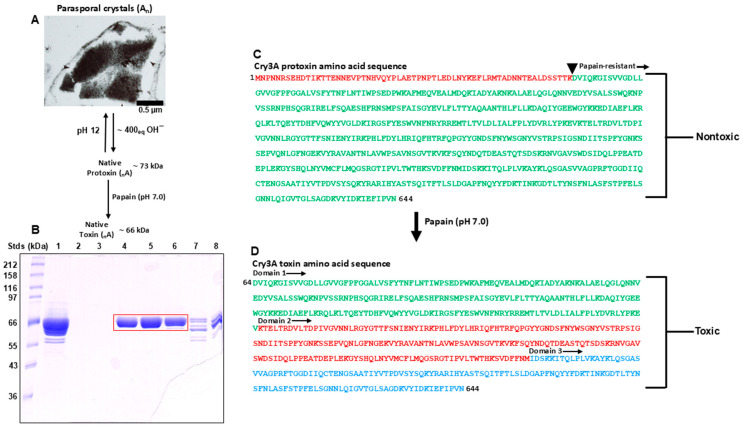
Annotated amino acid sequences of the Cry3A protoxin and toxin deduced from the nucleotide sequence of the Cry3A protoxin gene of Btt. (**A**) Parasporal crystals produced by the Btt strain UTD-001. (**B**) Purification of Cry3A toxin by anion exchange and gel filtration chromatography. Lane 1 represents toxin before chromatography and lanes 4–6 outlined in red represent toxin after chromatography. (**C**) Amino acid sequence of the protoxin (^1^MN^644^), which is not toxic, and the toxin (^64^DN^644^) embedded in the protoxin. The red letters in Figure 1C = N-terminal peptide ^1^MK^63^ which is released by papain cleavage (black arrowhead at ^63^KD^64^). The green letters = papain-resistant polypeptide (^64^DN^644^, black arrow pointing downstream) which is toxic. (**D**) Cry3A toxin showing three domains: Domain 1 (green letters, ^64^DV^290^); Domain 2 (red letters, ^291^KM^500^), Domain 3 (blue letters, ^501^IN^644^).

Figure 1 summarizes the behavior of purified Cry3A protoxin and toxin in solution. The complex of parasporal crystals (A_n_) produced by Btt during sporulation is composed of many single repeating subunits that are dissociated (within minutes) in native conformation (_n_A) by mild alkali titration (Figure 1A). A subunit functionally acts as a protoxin (^1^MK^644^, MW = 72.980 kDa) and is composed of an embedded toxin ^64^DN^644^ (Figure 1C, green letters) and a nontoxic N-terminal segment ^1^MK^63^ (Figure 1C, red letters) that is cleaved at ^63^KD^64^ by papain (Figure 1C, black arrowhead). The remaining polypeptide toxin (^64^DN^644^, MW = 66.412 kDa, green letters) is papain-resistant (see black arrow pointing downstream). Purification of the native Cry3A toxin was accomplished by anion exchange and gel filtration chromatography (Figure 1B). Lane 1 represents toxin before chromatography and lanes 4–6 outlined in red represent toxin after chromatography. The molecular weight of toxin and protoxin is based on the amino acid sequence of each molecule. The first reported crystal structure of Cry3A [18] is topologically like other Cry toxins including Cry1Aa and Cry4Ba [21,22] whose host range includes moths and mosquitoes, respectively. The structural model consists of 644 residues (^1^MN^644^). Unfortunately, the amino acid sequence (PDB 1DLC) published previously is that of protoxin, not toxin (^64^DN^644^, 581 residues) as reported in the present study. The fact that the single N-terminal residue methionine is present in quantitative yield indicates that the Cry3A protoxin is an intact product of translation. For clarification, the Cry3A toxin is presented in relationship to its precursor, the Cry3A protoxin (^1^MN^644^, Figure 1C). Protoxin per se is not toxic. Upon ingestion, the N-terminal segment of the protoxin (^1^MK^63^, red letters) is cleaved by proteases in the blind midgut of fourth-instar larvae, generating active toxin (^64^DN^644^, Figure 1D) which is transferred to the ventriculus where it binds to its attendant receptor, killing the insect. Papain activation in vitro at pH 7.0 releases the toxin (Figure 1B,D, MW ~66 kDa) from the protoxin. The activated Cry3A toxin (_n_A) most probably binds specifically to a cadherin GPCR, like those described for the Cry1Ab and Cry4B toxins [11,12], resulting in cell death. The Cry3A toxin itself is composed of three domains (Figure 1D): Domain 1 (green letters, ^64^DV^290^), an α-helical bundle of seven helices; Domain 2 (red letters, ^291^KM^500^), three antiparallel β sheets packed around a hydrophobic core; Domain 3 (blue letters, ^501^IN^644^), a sandwich of two antiparallel β sheets.

### 3.2. Cloning and Expression of the cry3a Gene

**Figure 2:**
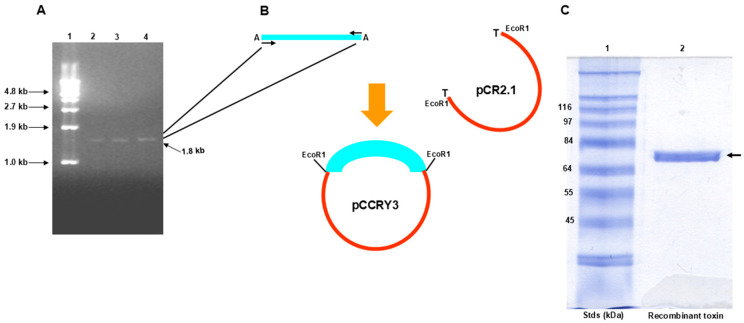
Cloning and expression of the *cry3a* gene. (**A**) Ethidium bromide-stained agarose gel of a 1.8 kb PCR product (lanes 2–4) from which the coding region of the *btr3a* gene was cloned and expressed. Lane 1 contains a ladder of markers. (**B**) The TA cloning vector used was pCR2.1 to produce the recombinant pCCRY3 plasmid containing the *btr3a* coding sequence. (**C**) Gene expression of the *btr3a* gene by transformed *E. coli* BL21 cells produced a protein (arrow, lane 2) that migrated to a position between markers 64 kDa and 84 kDa (lane 1) on a Coomassie blue-stained SDS-PAGE gel.

Figure 2 illustrates the cloning of the coding region (1743 nucleotides) for the Cry3A toxin gene from a 1.8 kb PCR product of UTD-001 (EtBr-stained gel, Figure 2A) into the TA cloning vector pCR2.1 to produce the pCCRY3 plasmid (Figure 2B). The gene sequence reveals that the coding region lies between ^190^*gat*^192^ (the first codon), which encodes aspartic acid, and ^1930^*aat*^1932^ (the last codon) which encodes asparagine. Alas, the nucleotide sequence first reported [23] is that of protoxin, not toxin. Expression of the purified *btr3a* gene in transformed *E. coli* BL21 cells resulted in a single protein (arrow, lane 2, Figure 2C) that was quite effective in killing fire ants. Papain treatment of the protein rendered it suitable for recrystallization.

### 3.3. Toxicity of Recombinant Cry3A Toxin to Worker Ants

**Figure 3:**
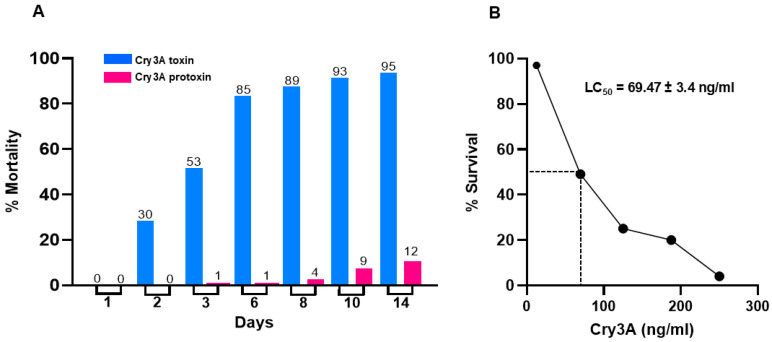
Toxicity of soluble recombinant Cry3A toxin to worker ants. (**A**) Purified recombinant toxin was administered to RIFA workers at a concentration of 200 µg/mL. The bioassays were performed under laboratory conditions for a period of 14 days during which % mortality was recorded daily. Approximately 95% of the ant population was killed by the recombinant toxin by day 14 (blue bars). Protoxin was ineffective (pink bars). (**B**) The LC_50_ value for Cry3A against worker ants is 69.47 ± 3.4 ng/mL. The LC_50_ value for native toxin is 70.04 ng/mL. Bioassay results were analyzed using GraphPad 10.4.1 (627) and represent the mean ± SD of six independent experiments replicated five times. See Section 2 for experimental details.

The toxicity of the recombinant Cry3A toxin is summarized in Figure 3. The purified recombinant toxin (200 µg/mL) was highly toxic to worker fire ants (blue bars, Figure 3A), killing 30% of the population within 2 days and 95% within 14 days. Notably, native protoxin (pink bars, Figure 3A) had minimal effect, as was true for whole spore/parasporal crystal mixtures, papain and sucrose). The LC_50_ value for recombinant Cry3A toxin on worker ants (Figure 3B) was 69.47 ng/mL. The LC_50_ value for native toxin was 70.04 ng/mL.

## 4. Discussion

Not since the first Bt-based insecticide, Sporeine, was produced in France in 1938, have any subspecies of Bt been reported to control fire ants, nor are there any Bt products commercially available for such purpose. What is needed is a biocidal construct that is effective in controlling populations of the fire ant yet is not toxic to non-target organisms. Such a biocide would have broad applicability in agriculture, the environment and public health. A Bt toxin effective in killing fire ants would be especially desirable. Certainly, the Cry3A toxin of Btt is a potential candidate. The laboratory studies reported here demonstrate that soluble recombinant Cry3A toxin (200 µg/mL) is incredibly active (Figure 3A) against the RIFA (LC_50_ = 69.47 ng/mL, Figure 3B), whereas protoxin and whole spore/crystal mixtures are not. Also, preliminary field experiments using soluble native toxin-coated, orange-flavored sugar cookie crumbs at the same concentration dramatically reduced ant populations in mound communities. Whole spores and crystals blended with cookie crumbs were ineffective, as were Cry4B (mosquito toxin) and Cry1Ab (moth toxin), pointing out the selectivity and specificity of the Cry3A toxin.

Fire ants are omnivorous—a large portion of their diet comprises invertebrates which the fire ant stings and kills. They also feed on dead animals and plant tissues. They are attracted to sugars, citrus, certain amino acids, various ions in solution and to some oils containing polyunsaturated fatty acids. Worker ants consume only liquid foods, and nearly half of the resources returned to the nest are in liquid form, which is slowly fed by trophallaxis to other workers, nurses and from nurses to larvae. Undissolved solids greater than 0.88 µm are screened from liquid in the pharynx of the worker fire ant and cannot be ingested. The solids accumulate in the buccal region as pellets and are later expelled to feed fourth-instar stage larvae, which can consume particles as large as 45 µm. Because solid food may be used by the mature larvae but not the workers, solids from the field move to these larvae more quickly and directly than liquid foods. After processing by the fourth-instar larvae, the resulting liquified foods are taken in by the queen and younger larvae also through trophallaxis. Because worker ants transport foods containing poisons and toxins back to the colony and distribute them throughout the colony, food composition and particle size are important considerations when constructing and designing insecticides to kill fire ants. A masterly and detailed account of the biology and behavior of the fire ant is contained in references [24,25].

Fire ants selectively filter objects such as bacterial spores and parasporal crystals away from the oral cavity, preventing entry of these items into the digestive system. So, it is important that Cry3A solids be limited to 0.88 µm or less to allow the fire ant to effectively ingest the poison. Soluble Cry3A toxin which can be derived from papain treatment of solubilized parasporal crystals (Figure 1) or from a recombinant construct (Figure 2) could be formulated as a sprayable liquid or a powder, e.g., fine particles coated with the toxin, to circumvent the size-exclusion defense mechanism of the fire ant. In any event, the Cry3A toxin prepared properly is a potential candidate for fire ant control. I believe that this toxin can be custom designed and exploited as an effective and efficacious product for controlling fire ants. Field trials using different bait formulations would be a worthwhile undertaking.

## 5. Conclusions

This study is the first to demonstrate that the Cry3A toxin of Btt kills the RIFA. The toxin is embedded in the parent protoxin and is cleaved from the protoxin enzymatically with papain. Both native and recombinant Cry3A toxins are equally effective in killing the fire ant.

## Data Availability

The original contributions presented in this study are included in the article. Further inquiries can be directed to the corresponding author.
